# Cardiac surgery in adults with congenital heart disease: an African perspective

**DOI:** 10.1186/1749-8090-10-S1-A323

**Published:** 2015-12-16

**Authors:** Michael A Long, Stephen C Brown, Francis E Smit, Sharon L Rossouw

**Affiliations:** 1 Department of Cardiothoracic Surgery, Faculty of Health Sciences, University of the Free State, PO Box 339 (Internal box G32), Bloemfontein, 9300, South Africa; 2 Department of Paediatric Cardiology, Faculty of Health Sciences, University of the Free State PO Box 339 (Internal box G32), Bloemfontein, 9300, South Africa

## Background/Introduction

A paucity of data exists on the spectrum and outcome of adult patients undergoing congenital heart surgery (CHS) on the African continent.

## Aims/Objectives

This study was undertaken to understand the local disease profile and needs of this patient group and so to facilitate planning for future provision of cardiac services.

## Method

A retrospective chart review was undertaken of all consecutive adult patients (≤ 18 years) undergoing CHS in a single African tertiary care hospital between October 1995 and January 2015. Patients and operative outcomes were described using the Society of Thoracic Surgeons CHS database form.

## Results

A total of 220 operations were performed in 209 patients (45% male). Mean age at surgery was 30,1 ± 10,9 years. Preoperative diagnostic cardiac catheterization was performed in 86,3% of patients. The most common lesions according to primary diagnostic category were as follows: Septal defects (43,6%), Right heart lesions, including Conduit failure (23,7%), Left heart lesions (10,5%) and Thoracic arteries and veins (8,6%). Single ventricle lesions comprised 2,7% of diagnoses. Fifty-four percent of patients presented in the moderate or complex Bethesda diagnostic classes. Preoperative risk factors were present in 19,1% of patients with endocarditis, renal dysfunction and severe pulmonary hypertension the most frequent. Reoperations constituted 28,6% of procedures performed. Right ventricle to pulmonary artery conduit placement constituted 50,8% of the reoperations. Overall operative mortality was 1,8% (n = 4) with 4,8% (n = 3) mortality in the reoperation group. Postoperative complications occurred in 26,8% of patients. The mean Aristotle Basic Score was 6,2 ± 2.4.

## Discussion/Conclusion

Surgical treatment is feasible in the African context with low mortality and acceptable morbidity in spite of limited resources. Our patient profile was similar to that reported in a recent multicentre European series. Our utilization of diagnostic cardiac catheterization seemed excessive. More than half of our patient group will require long-term specialized care.

